# MAGE-A inhibit apoptosis and promote proliferation in multiple myeloma through regulation of BIM and p21^Cip1^

**DOI:** 10.18632/oncotarget.27488

**Published:** 2020-02-18

**Authors:** Anna Huo-Chang Mei, Kaity Tung, Jessie Han, Deepak Perumal, Alessandro Laganà, Jonathan Keats, Daniel Auclair, Ajai Chari, Sundar Jagannath, Samir Parekh, Hearn Jay Cho

**Affiliations:** ^1^Tisch Cancer Institute, Icahn School of Medicine at Mt. Sinai, New York, NY, USA; ^2^Department of Genetics and Genomic Sciences, Icahn School of Medicine at Mt. Sinai, New York, NY, USA; ^3^Institute for Next Generation Healthcare, Icahn School of Medicine at Mt. Sinai, New York, NY, USA; ^4^Translational Genomics Research Institute, Phoenix, AZ, USA; ^5^The Multiple Myeloma Research Foundation, Norwalk, CT, USA

**Keywords:** MAGE-A3, multiple myeloma, apoptosis, cell cycle regulation, DNA repair

## Abstract

The type I Melanoma Antigen Gene (MAGE) A3 is a functional target associated with survival and proliferation in multiple myeloma (MM). To investigate the mechanisms of these oncogenic functions, we performed gene expression profiling (GEP) of p53 wild-type human myeloma cell lines (HMCL) after MAGE-A knockdown, which identified a set of 201 differentially expressed genes (DEG) associated with apoptosis, DNA repair, and cell cycle regulation. MAGE knockdown increased protein levels of pro-apoptotic BIM and of the endogenous cyclin-dependent kinase (CDK) inhibitor p21^Cip1^. Depletion of MAGE-A in HMCL increased sensitivity to the alkylating agent melphalan but not to proteasome inhibition. High *MAGEA3* was associated with the MYC and Cell Cycling clusters defined by a network model of GEP data from the CoMMpass database of newly diagnosed, untreated MM patients. Comparative analysis of CoMMpass subjects based on high or low *MAGEA3* expression revealed a set of 6748 DEG that also had significant functional associations with cell cycle and DNA replication pathways, similar to that observed in HMCL. High *MAGEA3* expression correlated with shorter overall survival after melphalan chemotherapy and autologous stem cell transplantation (ASCT). These results demonstrate that MAGE-A3 regulates Bim and p21^Cip1^ transcription and protein expression, inhibits apoptosis, and promotes proliferation.

## INTRODUCTION

The type I Melanoma Antigen GEne (MAGE-A, B, and C) family has emerged as a promising new class of therapeutic targets in cancer. They belong to the Cancer-Testis Antigen (CTAg) group of tumor-associated genes that were first characterized because they elicited immune responses in patients whose cancers express them [1]. As their name suggests, these genes are expressed in a broad range of human cancers but in normal tissues they are tightly restricted to developing germ cells and trophoblastic tissue. For these reasons, they have been investigated as targets for immunotherapeutic strategies such as tumor vaccines and transgenic T cell receptor-engineered lymphocytes [2–4], but their potential role in oncogenesis has only recently come under scrutiny. Type I MAGE partner with Really Interesting New Gene (RING) domain proteins to form ubiquitin ligase complexes. MAGE-RING complexes ubiquitinylate cancer-associated targets such as p53 and AMP-activated protein kinase to promote anchorage-independent growth and Warburg metabolism and to inhibit autophagy in laboratory models [5, 6]. Expression of MAGE-A genes and other CTAg in primary melanoma specimens was correlated with resistance to anti-CTLA-4 immune checkpoint therapy [7].

We showed that several type I MAGE, particularly MAGE-A3, C1, and C2, are commonly expressed in multiple myeloma (MM), a cancer of plasma cells, and are associated with proliferation and resistance to apoptosis [8, 9]. MAGE-A3 is expressed in about 35% of newly diagnosed MM and more that 75% of relapsed MM cases [9], which is substantially higher than in most other cancers; by comparison, MAGE-A3 is expressed in about 50% of non-small cell lung cancers, 36% of melanomas, and 20% of breast cancers [1]. Silencing of MAGE-A family members in either p53 wt or null (mutated and/or deleted) human MM cell lines (HMCL) by RNA interference (RNAi) resulted in rapid induction of intrinsic apoptosis, characterized by mitochondrial membrane depolarization, upregulation of pro-apoptotic BAX and cleavage/activation of Caspases 3 and 9, and decreased proliferation characterized by loss of entry into S phase [9]. In this setting, upregulation of BAX and decreased proliferation was only observed in p53 wt HMCL, indicating that MAGE regulates at least one other p53-independent pathway. MAGE expression has been associated with poor clinical outcome in MM, and correlated with resistance to chemotherapy in clinical trials and laboratory models, including resistance to panobinostat in MM patients [10–13]. These results support the hypothesis that MAGE-A are oncogenic targets that inhibit apoptosis and promote proliferation in myeloma cells.

We report here on laboratory models and translational studies of gene expression profiling and patient outcome data that illuminate two proximal mechanisms of MAGE-A regulation of apoptosis and proliferation in multiple myeloma. Gene expression profiling (GEP) of p53 wt HMCL after MAGE-A knockdown demonstrated a set of 201 differentially expressed genes (DEG) enriched for genes involved in apoptosis, DNA damage repair, and cell cycle regulation. Corresponding analysis showed that MAGE knockdown increased protein levels of the BH3-only Bcl-2 family member BIM, a key initiator of intrinsic apoptosis in hematopoietic lineages, and of the endogenous cyclin-dependent kinase (CDK) inhibitor p21^Cip1^. Silencing of MAGE-A in HMCL increased sensitivity to the alkylating agent melphalan but not to proteasome inhibition. Supervised analysis of GEP data from newly diagnosed, untreated MM patients based on *MAGEA3* expression revealed significant associations with co-expression of other CTAg genes and with cell cycle and DNA replication pathways. High *MAGEA3* expression correlated with worse clinical outcome, particularly in the context of autologous stem cell transplantation (ASCT) with melphalan conditioning chemotherapy. These results demonstrate that MAGE-A regulates Bim and p21^Cip1^ to inhibit apoptosis and promote cell cycle progression, and this activity contributes to survival, resistance to chemotherapy, and proliferation in p53 wt MM cells.

## RESULTS

### Silencing of MAGE-A results in pro-apoptotic gene expression program

**Figure 1:**
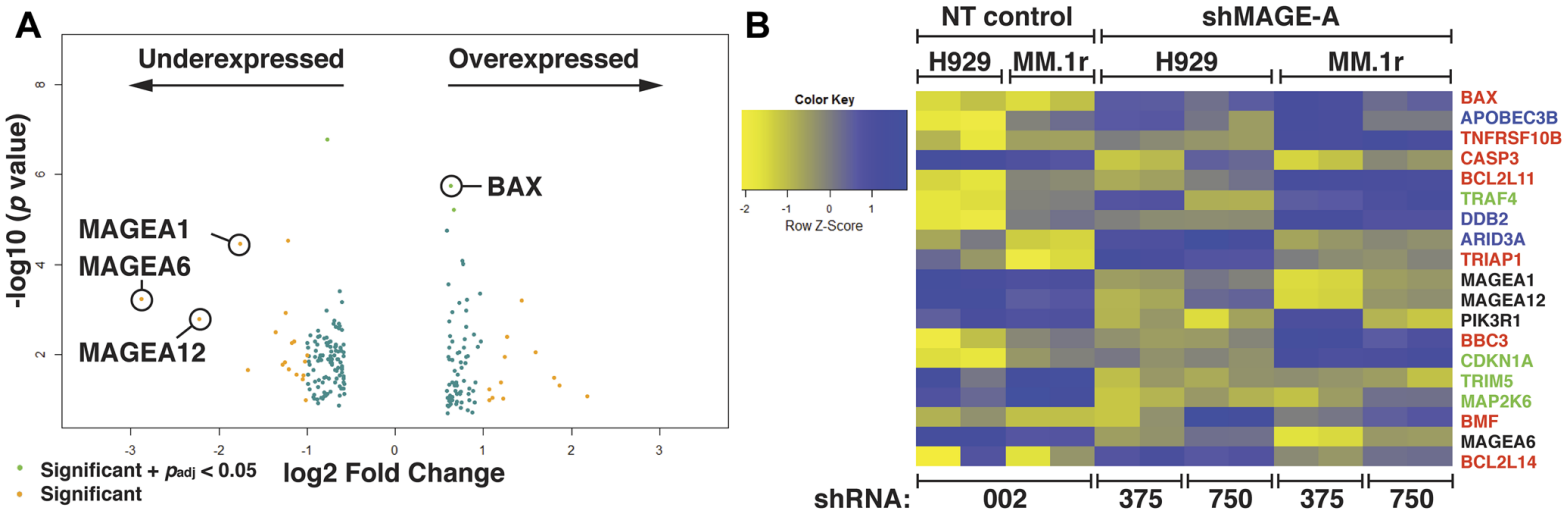
Gene expression profiling of HMCL after silencing of MAGE-A. (**A**) Volcano plot of 201 gene set with minimum 2-fold change in expression enriched from RNA seq data from MM.1r and H929 HMCL after silencing of MAGE-A by RNAi with two distinct shRNA lentiviral constructs compared to non-target control. Several MAGE-A family members are downregulated, likely reflecting direct effect of shRNA construct. Pro-apoptotic Bcl-2 genes such as BAX are among the significantly upregulated genes. *P*_adj_ calculated by multiple testing using the Benjamini-Hochberg method. (**B**) Heat map of top over- and under-expressed genes. Apoptosis-associated genes are depicted in red. DNA-binding and repair genes are depicted in blue. Cell cycle-associated genes are depicted in green. NT, non-target shRNA lenti (SHC002, denoted as 002. shMAGE-A, MAGE-A3-targeted shRNA lenti (TRCN0000128375 and TRCN0000129750, denoted as 375 and 750, respectively).

We previously reported that silencing of MAGE-A in HMCL resulted in apoptosis, and in p53 wt HMCL, decreased entry into S phase [9]. Functional RNAi screening of 34 p53 wild type (wt) or null HMCL support this finding, demonstrating that MM cells are highly dependent upon MAGE-A3 for survival (Supplementary Figure 1, Supplementary Tables 1 and 2 [14–16]). To interrogate the proximal mechanisms of these activities, we performed gene expression profiling (GEP) by RNA sequencing on MM.1r and H929 HMCL (both p53 wt) transduced with either of two MAGE-A3-targeting shRNA lentiviral constructs (TRCN0000128375 and TRCN0000129750, Sigma-Aldrich) which target distinct sequences of the MAGE-A3 transcript and were previously shown to efficiently knock down MAGE-A3 RNA and protein levels and induce apoptosis [9]. These constructs also silence mRNA and protein expression of MAGE-A1, A3, A4, and A6, reflecting the high degree of sequence homology and potentially shared functions among MAGE-A [17, 18], but MAGE-C1 was not affected. MAGE-A3 is the most common A family member expressed in HMCL and primary MM, but given the promiscuous activity of the shRNA constructs, we collectively refer to them in these experiments as MAGE-A. Cells transduced with a scrambled, non-target lenti construct (SHC002) served as controls. Cells were harvested after reduction of MAGE-A3 protein was detected but before the onset of apoptosis (48 hrs for MM.1r, 72 hrs for H929), and total RNA was extracted for RNAseq. Comparative analysis of GEP between MAGE-A-silenced and control-transduced cells enriched a set of 201 differentially expressed genes (201 DEG, *p*_adj_ < 0.05, Figure 1A). Among these were several MAGE-A family members (A1, A6, and A12), likely reflecting the direct effect of the shRNAs on homologous transcripts. Of the top DEGs, eight were BH3-only Bcl-2 family members (BAX, BCL2L11/BIM, BBC3/PUMA, BMF, BCL2L14/BCLG) or apoptotic pathway genes (TNFRSF10B/TRAILR2, CASP3, TRIAP1) (Figure 1B). Four of the top DEGs were cell cycle regulation genes (TRAF4, CDKN1A/p21^CIP1^, TRIM5, MAP2K6/MEK6) and three were DNA binding/damage repair genes (APOBEC3B, DDB2, ARID3A). Functional enrichment analysis of the 201 DEG set showed strong associations with cancer, p53 activity, and apoptosis (Table 1). Some of these pathway members such as BIM and PUMA had no previous association with MAGE-A3.

**Table 1:** Top 10 pathways associated with 201 DEG by gene set enrichment analysis in HMCL after MAGE-A knockdown by RNAi

Term	Overlap	*p*-value	Z-score	Genes
TP53 regulates transcription of cell death genes_Homo sapiens_R-HAS-5633008	5/43	6.88E-05	-1.95902	TNFRSF10B; BAX; TRIAP1; BCL2L11; BBC3
Intrinsic pathway for apoptosis_Homo sapiens_R-HAS-109606	5/42	6.13E-05	-1.9168	BCL2L11; CASP3; BAX; BMF; BBC3
BH3-only proteins associated with and inactivate anti-apoptotic Bcl-2 members_Homo sapiens_R-HAS-111453	3/8	5.4E-05	-1.55628	BCL2L11; BMF; BBC3
Protein repair_Homo sapiens_R-HAS-5676934	3/6	1.96E-05	-1.50801	MSRB2; MSRB3; MSRB1
Programmed cell death_Homo sapiens_R-HSA-5357801	7/166	0.001474	-2.12833	BCL2L11; CASP3; BAX; TNFRSF10B; CTNNB1; BMF; BBC3
Apoptosis_Homo sapiens_R-HAS-109581	7/163	0.001327	-2.11426	BCL2L11; CASP3; BAX; TNFRSF10B; CTNNB1; BMF; BBC3
Transcriptional regulation by TP53_Homo sapiens_R-HSA-3700989	10/348	0.00287	-2.23051	CDKN1A; SESN2; BAX; TNFRSF10B; ARID3A; TRIAP1; BCL2L14; TNRC6A; BBC3; DDB2
TP53 regulates transcription of genes involved in Cytochrome C release_Homo sapiens_R-HAS-6803204	3/19	0.000861	-1.81256	BAX; TRIAP1; BBC3
Cholesterol biosynthesis_Homo sapiens_R-HSA-191273	3/23	0.001527	-1.84964	ARV1; IDI2; LSS
Signaling by WNT in cancer_Homo sapiens_R-HSA-4791275	3/34	0.004758	-1.99323	FZD4; FZD8; CTNNB1

### MAGE-A knockdown results in increased BIM and p21^Cip1^ proteins

**Figure 2:**
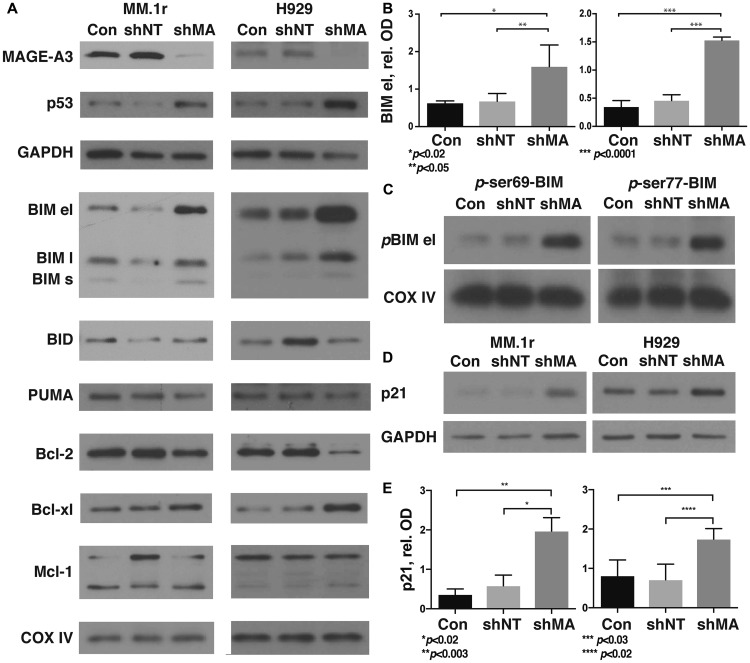
Silencing of MAGE-A in HMCL results in stabilization of phosphorylated BIM and p21^Cip1^. MM.1r and H929 were transduced with MAGE-A3-specific lentiviral shRNA constructs or controls as described, and heavy membrane preps (A, C) were probed for expression of Bcl-2 family proteins. (**A**) Western blot of heavy membrane preps for Bcl-2 family proteins. MAGE-A3 knockdown and increased p53 protein were confirmed by western blot of whole cell lysate. COXIV, load control, representative of all blots. (**B**) Optical densitometry of western blots for BIM el as in (A) demonstrates significantly higher levels of this protein after MAGE-A silencing compared to controls. Data was pooled from five replicates, representing 13–15 data points each. Error bars, standard error of the mean. (**C**) Western blot of heavy membrane preps for p-ser69 and p-ser77 Bim el demonstrate stabilization of the phosphorylated protein after MAGE-A knockdown. (**D**) Whole cell lysates were probed by western blot for p21^Cip1^. (**E**) Optical densitometry of western blots for p21^Cip1^ as in (D) demonstrates significantly higher levels after MAGE-A silencing. Data pooled from five replicates, representing 15 data points each. Con, Untreated control cells. shNT, non-target shRNA lentiviral construct. shMA, MAGE-A3 targeted lentiviral shRNA construct TRCN0000128375.

We assessed protein expression of Bcl-2 family members and p21^Cip1^ in MAGE-A lenti shRNA transduced HMCL and controls. As previously demonstrated, knockdown of MAGE-A resulted in stabilization of p53 protein levels (Figure 2A, Supplementary Table 3) [9]. The 201 DEG suggested focus on the so-called “death activator” BH3-only proteins which initiate intrinsic apoptosis (BIM, BID, and PUMA), as well as BMF and BCLG. We also examined Mcl-1, which was shown to play a critical role in bortezomib-induced apoptosis [19], and on anti-apoptotic Bcl-2 and Bcl-xl. MAGE-A shRNA-transduced (TRCN0000128375) and control cells were harvested prior to the onset of apoptosis and heavy membrane preparations were isolated to assess membrane-associated Bcl-2 family proteins. Western blotting of heavy membrane preparations demonstrated significantly increased levels of the extra-long isoform of BIM (el, Figure 2A and 2B, Supplementary Table 3), whereas BID and PUMA levels appeared unperturbed. Full-length and cleaved Mcl-1 demonstrated variable expression under these conditions. Bcl-2 appeared slightly decreased under these conditions but this did not achieve statistical significance, and Bcl-xl, BMF, and BCLG were essentially unchanged.

BIM protein stability is governed by several post-translational modifications. Phosphorylation at Ser69 and Ser77 by ERK1/2, JNK, and other members of the MAP kinase family target the protein for ubiquitinylation and proteasomal degradation [20, 21]. Silencing of MAGE-A increased levels of both phospho (*p*)-Ser69- and *p*-Ser77-Bim, indicating that MAGE-A does not affect kinase activity in this setting (Figure 2C). These results suggest that phosphorylated Bim is either not being Ub or not being degraded in the absence of MAGE-A.

P21^Cip1^ is an endogenous inhibitor of cyclin D1-CDK4/6 activity that inhibits passage through the early G1 checkpoint of the cell cycle. Dysregulation of the early G1 checkpoint is a hallmark of MM patients characterized by *t*(11;14), which places *CCND1* (cyclin D1) under the control of the Ig heavy chain promoter [22]. Western blotting of whole cell lysates demonstrated that p21^Cip1^ protein levels were significantly increased after depletion of MAGE-A (Figure 2D and 2E).

To assess the role of p53, we performed shRNA knockdown experiments in p53 null HMCL RPMI-8226 (homozygous for p53 p. Glu285Lys) and PCNY1 (17p-; p53 p. Arg248Gly). Silencing of MAGE-A in these cells resulted in apoptosis as previously described [9], but did not affect protein expression of BIM or p21 (Supplementary Figure 1), indicating that MAGE regulate at least one p53-independent mechanism of apoptosis and/or proliferation.

### MAGE-A silencing increases sensitivity to melphalan

**Figure 3:**
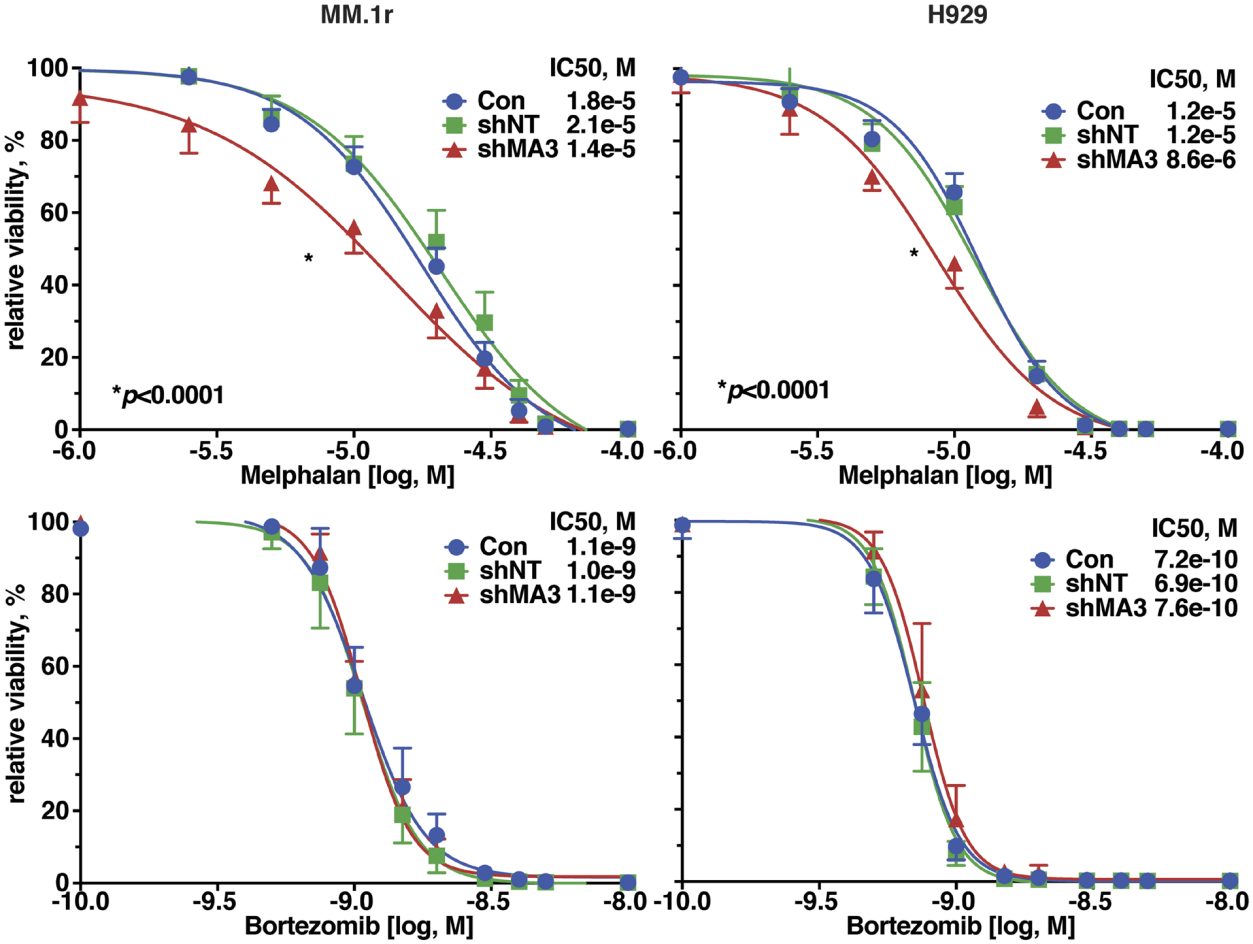
Silencing of MAGE-A in HMCL increased sensitivity to melphalan-induced apoptosis. MM.1r and H929 cells were transduced with MAGE-A3-specific lentiviral shRNA constructs or controls as described and incubated with increasing concentrations of melphalan (top graphs) or bortezomib (bottom graphs) and cell viability was assessed after 24 hrs by Cell TiterGlo assay.

We previously demonstrated that MAGE-A was associated with resistance to panobinostat, but not lenalidomide, in clinical trials and laboratory models [10]. To further investigate the impact of MAGE-A on chemotherapy-induced apoptosis, we silenced MAGE-A expression in MM.1r and H929 HMCL by transduction of MAGE-A lentiviral shRNA construct as previously described. Lentivirus-transduced cells were incubated for 48 (MM.1r) or 72 (H929) hrs to capture live cells with loss of protein expression of MAGE-A3 but before the onset of apoptosis. We then treated the cells with two chemotherapy agents commonly used in MM; melphalan, an alkylating agent, or bortezomib, a proteasome inhibitor, and assessed viability by quantitation of ATP. Loss of MAGE-A resulted in significantly decreased LD50 for melphalan, whereas no significant change in LD50 for bortezomib was observed (Figure 3).

### Expression of MAGEA3 in primary myeloma correlates with survival and is associated with cell cycle regulation and DNA repair

**Figure 4:**
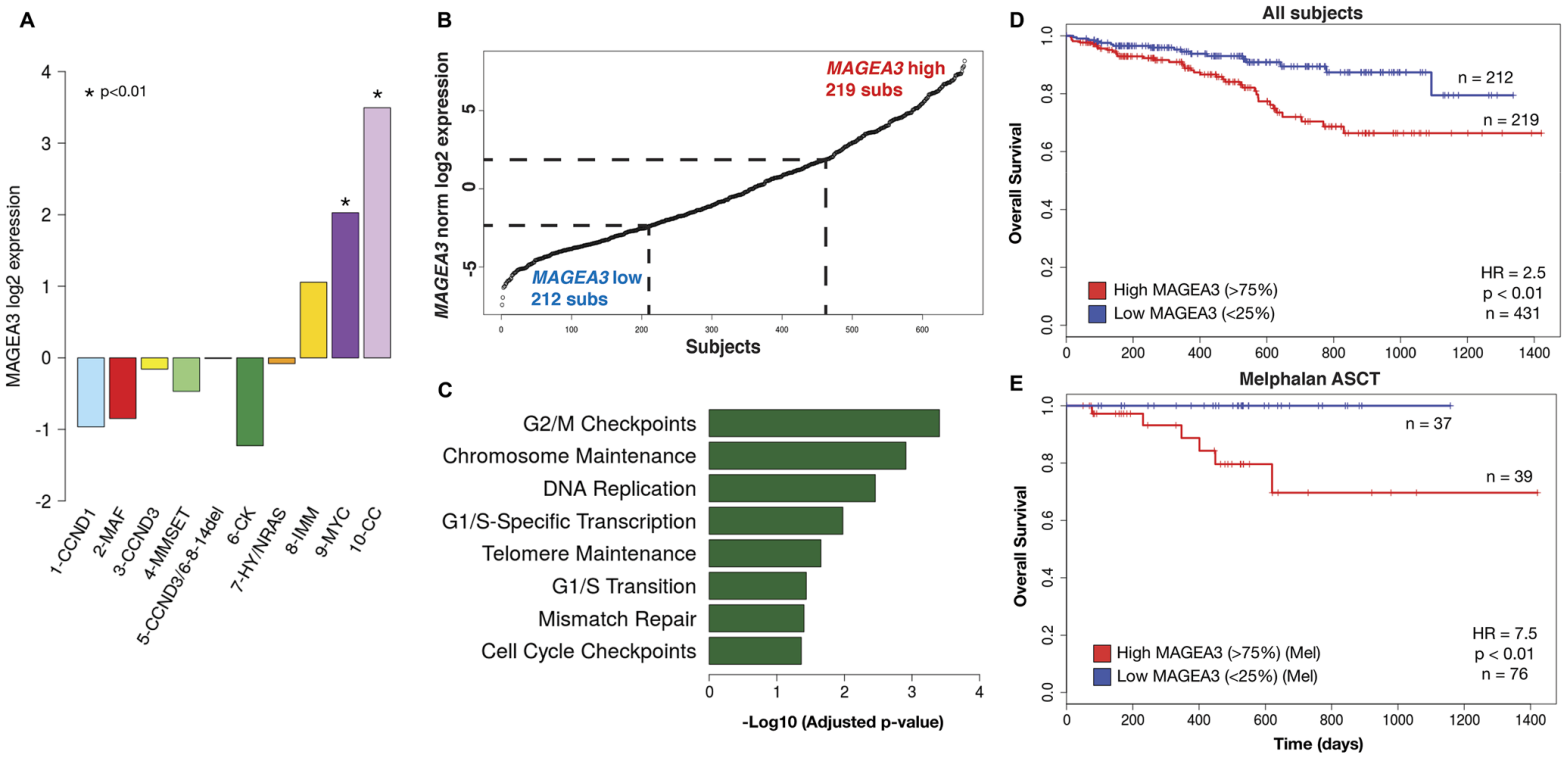
*MAGEA3* expression is associated with cell cycling, DNA replication/repair, and poor prognosis. (**A**) Comparison of average *MAGEA3* expression in patient subsets identified in MMNet analysis of the CoMMpass iA7 release^25^ demonstrated high *MAGEA3* associated with the MYC and CC clusters. (**B**) Subjects from the CoMMpass iA9 release dataset were stratified by *MAGEA3* expression and differential gene expression was assessed between the highest and lowest quartiles. (**C**) Gene set enrichment analysis of differentially expressed genes demonstrated strong associations with cell cycling, DNA replication and repair pathways associated with high *MAGEA3*. (**D**) Kaplan-Meier projection of overall survival in *MAGEA3* low and high quartiles of all subjects shows a significant association with shorter overall survival in the *MAGEA3* high quartile compared to low. (**E**) Subset analysis of *MAGEA3* high and low quartiles in patients who underwent melphalan conditioning and autologous stem cell transplantation for consolidation showed significantly worse overall survival associated with the *MAGEA3* high quartile.

MAGE-A3 is detected in approximately 35% of newly diagnosed MM cases [9]. Laganà *et al.* recently published a novel network analysis of tumor gene expression profiling data from the CoMMpass dataset iA7 release, comprising approximately 450 newly diagnosed, untreated MM patients [23]. This analysis, which incorporated co-expression network annotation and correlation with genomic alterations and clinical characteristics, identified ten clusters of MM patients. We compared average *MAGEA3* expression in each of these clusters, and two of these, one characterized by the *t*(8;14) translocation and cMyc overexpression (MYC) and a second characterized by a cell cycle gene expression signature (CC), featured significantly higher *MAGEA3* expression compared to the other eight (Figure 4A). These two clusters accounted for about 13% of patients and were also associated with copy number alterations, and the CC cluster was also associated with increased mutational burden. To investigate the significance of *MAGEA3* expression in new MM, we interrogated the CoMMpass database iA9 release, which is updated with GEP data from more than 650 newly diagnosed, untreated MM patients, based on *MAGEA3* gene expression. We performed comparative analysis of the highest 25% (219 subjects) of *MAGEA3* expression versus the lowest 25% (212 subs, Figure 4B). This analysis enriched a set of 6748 differentially expressed genes (*p* < 0.05, Table 2). Thirteen of the top 25 differentially expressed genes were X-linked, including eleven Cancer-Testis Ag genes (*MAGEA, B families*, *SSX1*, *CSAG1, CTAG1, 2, PAGE5, XAGE3, 5, and DDX53*). Several non-coding RNAs (*LINCs, TEX41*) and non-X-linked genes that are normally expressed in testis and/or associated with cancer were also among the top 25 DEGs. Gene set enrichment analysis demonstrated significant associations with cell cycle regulation and with DNA replication and repair pathways (Figure 4C). Clinical outcome based on *MAGEA3* expression demonstrated that there was no significant difference in progression-free survival, but the *MAGEA3* high quartile demonstrated worse overall survival compared to *MAGEA3* low (HR = 2.5, *p* < 0.01. Figure 4D). This survival difference was even greater when analysis was restricted to subjects who received melphalan chemotherapy and ASCT (HR = 7.5, *p* < 0.01. Figure 4E).

**Table 2:** Top 25 differentially expressed genes associated with the highest MAGE-A3 expressing quartile in the CoMMpass iA9 release

Gene Name	logFC	Adj. *p*-val	X-linked	Testis expressed	Cancer associated
**MAGEA6**	5.210435881	5.65E-90	X	X	X
GABRA3	4.386472649	1.25E-77	X		
LINC01518	3.643152976	1.16E-57		X	
**CSAG1**	3.640097632	1.26E-57	X	X	X
**MAGEA12**	3.907762103	9.04E-55	X	X	X
**PAGE5**	3.872984758	1.10E-52	X	X	X
**SSX1**	3.879821731	4.35E-48	X	X	X
LINC01287	3.180678435	2.23E-45		X	
DUXAP9	3.188513069	2.41E-45			
CHRM3	3.492811115	3.86E-42			
**MAGEA1**	3.69704424	2.59E-41	X	X	X
TEX41	3.215802797	8.73E-41		X	
NA	2.582806714	1.39E-40	X		
GABRG2	3.462428666	1.51E-40			
**XAGE5**	2.863938516	3.76E-40	X	X	X
SLCO1A2	3.293803231	2.89E-39			
**CTAG2**	3.561958381	5.58E-39	X	X	X
**XAGE3**	3.021355896	8.44E-39	X	X	X
BDKRB1	2.649197266	2.56E-38			
PKHD1	3.254813944	2.72E-38		X	
**DDX53**	2.941927628	7.01E-38	X	X	X
**MAGEB2**	3.26323396	1.18E-35	X	X	X
ADAMTS20	2.767539897	3.19E-35		X	
CEACAM16	2.75184892	8.35E-35			X
CYCSP6	2.385479068	1.18E-34			

Log Fold Change (logFC) and adjusted *p*-values (Adj. *p*-val) are listed. Cancer-Testis Antigen genes are in bold. X-linked genes, genes with relatively restricted expression in testis, and genes associated with cancer are identified by “X” in the respective columns.

## DISCUSSION

The results presented here support a central role for type I MAGE in survival, chemotherapy resistance, and proliferation in p53 wt MM cells. Gene and protein expression studies in HMCL after MAGE-A knockdown demonstrated significant increases in BAX [9] and BIM RNA and protein levels. Protein expression of the other death activators, BID and PUMA, or of other inducible and anti-apoptotic Bcl-2 family members was not significantly altered by loss of MAGE-A despite increased mRNA levels. Depletion of MAGE did not affect BIM phosphorylation, but phospho-BIM was increased under these conditions, indicating that further post-translational modification such as ubiquitinylation and subsequent proteasomal degradation did not occur. Therefore, MAGE-A appear to regulate BIM at both the transcriptional and post-translational levels.

P21^Cip1^ belongs to a family of CDK4/6 inhibitors that regulate passage through the early G1 checkpoint of the cell cycle and is a target gene of p53 transcriptional activity. P21^Cip1^ gene-deficient mice develop spontaneous tumors, and approximately 65% of these were of hematologic origin including B cell lymphomas [24]. Furthermore, dysregulation of cyclinD1-CDK4/6 activity promotes progression of disease in a subset of MM patients [22]. We previously showed that MAGE-A negatively regulate p53, and that decreased entry into S phase was observed after silencing of MAGE-A in p53 wt HMCL but not in p53 null cells [9]. The present study indicates that MAGE-A promotes proliferation in part through decreased p21 expression due to inhibition of p53. Neither BIM nor p21 protein levels appears to be affected by MAGE-A silencing in p53 null HMCL, but these cells still undergo apoptosis under these conditions, indicating that MAGE-A regulate at least one additional p53-independent survival pathway in myeloma cells. Multiple independent functional RNAi screens of both p53 wt and null cell lines demonstrated a strong dependency upon MAGE-A3 for survival [14–16], and loss of p53 through deletions and/or mutations are rare, estimated at less than 5% of newly-diagnosed MM [25]. Therefore, p53-dependent regulation of BIM and p21 are likely to be physiologically relevant in the majority of MM patients.

The analysis of differential gene expression in newly diagnosed patients based on high or low *MAGEA3* expression revealed two novel associations. First, a diverse array of CTAg genes are co-expressed with *MAGEA3*, suggesting coordinated dysregulation of these X chromosome genes in a subset of MM patients. Many of the CTAg loci are linked to CpG islands, and their expression appears to be principally governed by epigenetic events such as Cytidine demethylation [26, 27]. Genome wide methylation analysis of newly diagnosed MM specimens was predominantly characterized by hypomethylation, which may be a mechanism for broad overexpression of these genes in a subset of MM patients [28]. These results also agree with previous GEP and RT-PCR analyses which showed that expression of type I MAGE and other CTAg in primary MM specimens correlated with poor prognosis [11, 29]. Several of the CTAg genes detected in the CoMMpass analysis (MAGEA3, A6, A12, and CSAG1) were also in the CTAg gene set associated with resistance to anti-CTLA-4 therapy in melanoma [7]. The results presented suggest MAGE may contribute to resistance through inhibition of immunogenic (apoptotic) tumor cell death. Second, functional analysis of DEGs associated with MAGE-A3 revealed significant correlations with cell cycle progression, DNA replication, and DNA repair pathways. GEP analysis of HMCL after RNAi silencing of MAGE-A revealed another facet of their activity, as gene sets associated with p53-dependent and independent mechanisms of apoptosis, as well as DNA repair, were upregulated in agreement with our previous observations [9]. *MAGEA3* was also featured in network modules associated with cell cycling and MYC overexpression, reinforcing the association between proliferation and DNA repair [23]. Of note, MYC drives ongoing DNA damage, one of the hallmarks of clonal evolution in MM, through replicative stress during S phase [30].

We previously showed that MAGE-A expression was associated with short progression free survival in a clinical trial of panobinostat (a pan-HDAC inhibitor)-based chemotherapy, and that loss of MAGE-A sensitized HMCL to panobinostat but not lenalidomide, an immunomodulatory drug commonly used in MM [10]. In laboratory models of melanoma, MAGE-A recruited HDACs to p53 transcription sites and conferred resistance to etoposide, a topoisomerase inhibitor that induces DNA damage [12]. In this study, loss of MAGE-A increased sensitivity to melphalan-induced apoptosis, but not bortezomib. HDAC inhibitors such as panobinostat affect chromatin remodeling and transcriptional regulation, and alkylators and topoisomerase inhibitors induce double-stranded DNA breaks, whereas proteasome inhibitors and ImiDs do not directly interact with chromosomal DNA. These results, in conjunction with the GEP studies in patients and HMCL, reveal new associations among MAGE-A3, DNA replication and repair, and resistance to chemotherapy-induced apoptosis. Clinically, this is reflected in the significantly worse overall survival after melphalan-based ASCT in patients high *MAGEA3* expression.

**Figure 5:**
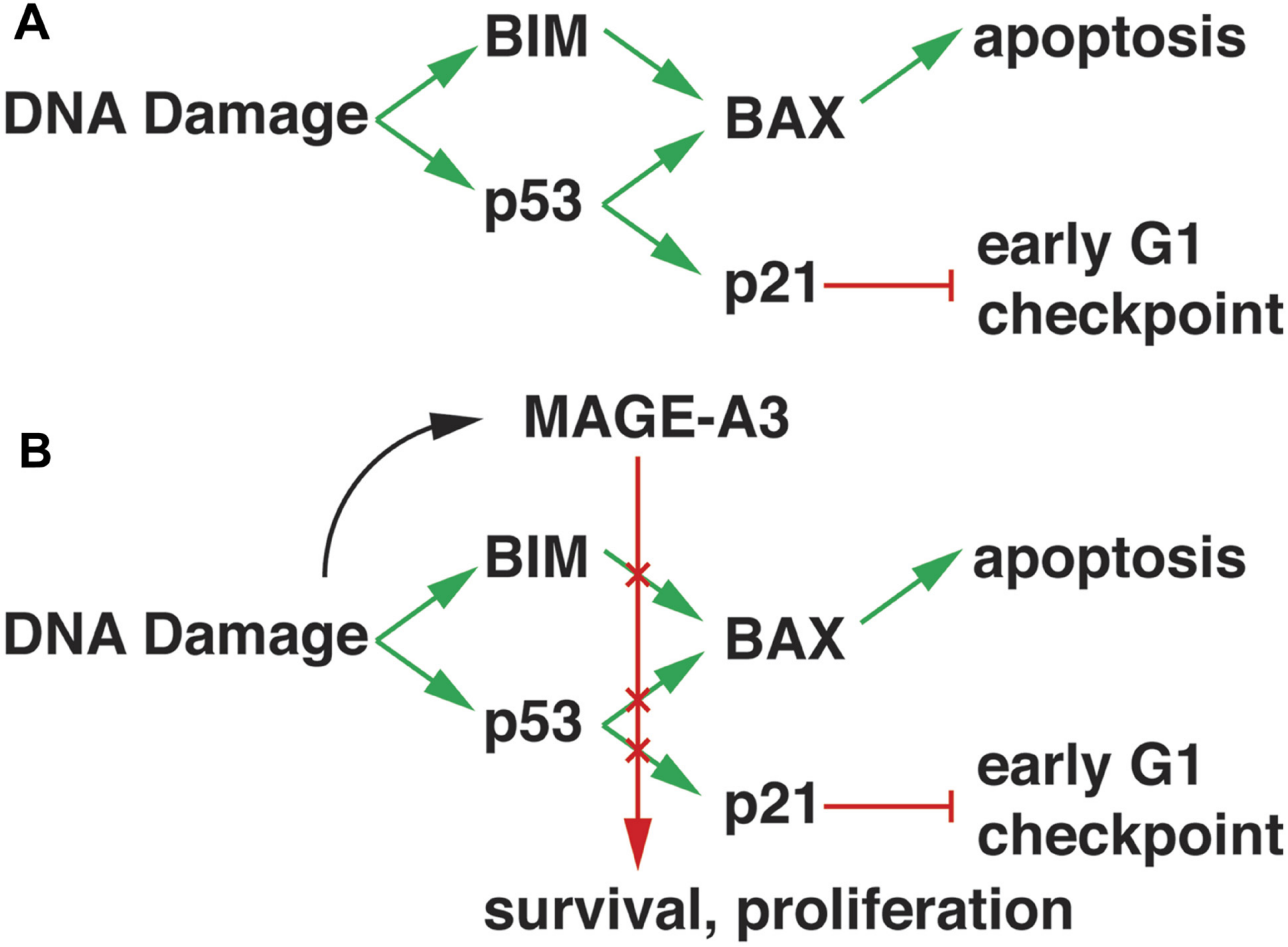
Model of MAGE-A3 activity in MM. (**A**) DNA damage response pathways promote BIM expression and p53 transcriptional activity, resulting in increased expression of BAX and p21^Cip1^. BIM displaces BAX from anti-apoptotic Bcl-2 family proteins, allowing it to translocate to mitochondrial membranes and initiate apoptosis. P21^Cip1^ inhibits cyclin D1/CDK4/6 activity, blocking progression through the early G1 checkpoint. (**B**) MAGE-A3/RING complex activity, possibly activated through interactions with DNA repair pathways, down-regulates BIM and p53 through transcriptional and post-translational mechanisms, resulting in survival and proliferation.

These results support a mechanistic model for MAGE-A3 oncogenic activity in p53 wt MM. Spontaneous mutations and double-stranded DNA breaks induced by chemotherapy such as alkylating agents would normally mobilize p53-dependent and independent DNA repair mechanisms that would induce cell cycle arrest through CDK inhibitors such as p21^Cip1^ and apoptosis via BIM and BAX (Figure 5A). However, in the presence of MAGE-A3, p53 is degraded, leading to decreased expression of BAX and p21^Cip1^. In addition, BIM transcription is decreased, and BIM protein is degraded, resulting in survival and proliferation (Figure 5B). Kap1, one of the known complex partners of MAGE-A3 in cancer cells, also functions in DNA repair (reviewed in [31]), but it is unclear if it is the sole partner in MM cells as it is typically restricted to the nucleus. BIM post-translational modification is a cytoplasmic activity, and it is possible that a distinct MAGE-A3 partner mediates this function. Alternatively, MAGE complexes may regulate expression of BIM-modifying enzymes at the transcriptional level. Further investigation is required to distinguish these mechanisms. There is a strong association with p53 in this setting, but apoptosis was also observed in p53 null HMCL (mutated and/or deleted) after MAGE-A silencing [9], supporting roles for p53-independent pathways in MAGE-A resistance to apoptosis. Therefore, MAGE-A3 expressing clones would have survival and proliferative advantages, which accounts for the strong associations with progression of disease and poor outcome, particularly in the context of melphalan ASCT.

The link between DNA replication and repair, inhibition of apoptosis, and proliferation is intriguing, as very little is known about the normal function of MAGE-A in germ cell development. Murine *Magea1* and *a3* were detected by *in situ* hybridization in developing mouse spermatogonia, where chromatin remodeling and DNA repair pathways are highly active during homologous recombination [32]. Similar expression of MAGE-A1 and -A4 was seen in normal human spermatogonia adjacent to primary germ cell tumors [33]. These results lead to the hypothesis that MAGE-A interact with DNA repair pathways in developing germ cells to inhibit apoptosis in the setting of genome-wide double stranded DNA breaks and resolutions. In support of this hypothesis, deletion of the *Magea* cluster in male mice (*Magea*^–/Y^) resulted in smaller testes and increased germ cell apoptosis at baseline and in response to nitrosurea, another alkylating agent [34]. This was characterized by stabilization of p53 protein and increased expression of Bax and p21^Cip1^, similar to our findings in myeloma. These DNA repair-linked functions may be subverted in tumor cells to allow survival in the setting of DNA-damaging chemotherapy agents. This function may also inhibit apoptosis in the setting of ongoing mutagenesis through MYC overexpression and aberrant DNA repair activity such as the APOBEC pathway [35, 36], providing a pivotal role for MAGE-A in clonal evolution that is a hallmark of relapse and chemotherapy resistance in MM [37, 38]. Finally, these results suggest that pharmacologic inhibition of MAGE-A activity in complex with Kap1 and other RING-domain partners may induce apoptosis directly and antagonize resistance to chemotherapy-induced apoptosis. MAGE-A inhibitors may have clinical application in MM and other MAGE-expressing cancers such as melanoma, lung, breast, and prostate cancers.

## MATERIALS AND METHODS

### Gene expression and survival analysis of CoMMpass dataset

The dataset included 660 samples from newly diagnosed patients in CoMMpass. Raw fastq files were aligned to the GRCh37 reference human genome and all annotation and gene models were based on Ensembl version 74. RNAseq fastq files were aligned to the reference genome using STAR (2.3.1z) [39]. Gene expression estimates (counts) were calculated using HT-Seq (0.5.3p3) [40]. We filtered gene expression data to include genes with cpm (counts per million) > 1 in at least 10% of the 660 samples. A total of 16,610 genes was retained. Filtered data was then normalized by TMM from the package edgeR and voom from the package Limma [41, 42]. We applied linear regression for gender and batch effect removal and kept residuals for further gene expression analysis, which was carried out by using the R package Limma.

The Kaplan-Meier survival estimate was fit using the function “survfit” from the R library “survival”. The *p*-values were calculated using the LogRank test as implemented in the R function “coxph”.

CoMMpass sequencing data is available through protected access on the dbGaP database (http://www.ncbi.nlm.nih.gov/gap) under accession number phs000748.

### RNA sequencing and data analysis

Total RNA was isolated using PureLink RNA Mini Kit (Life Technologies). We extracted poly A–selected RNA from MM.1r and H929 HMCL transduced with either of two MAGE-A3-targeting shRNA lentiviral constructs and scrambled control. In total, 100 ng of total RNA input was used to construct RNASeq libraries using TruSeq RNA Sample Preparation Kit v2 (Illumina) following the manufacturer’s instructions. Sequencing was done on a HiSeq 2500 System using 100 bases and paired-end read sequencing for scrambled and transduced constructs. Next, the reads with quality Phred scores more than 30 were aligned to reference sequence database (UCSC hg19), as well as RefSeq exons, splicing junctions, and contamination databases, including ribosome and mitochondria sequences using the Burrows–Wheeler Aligner (BWA). First, to compare the expression levels, the read counts of transcripts in each sample were normalized by leveling the total read count in each sample to the maximum of the read counts in all samples. Secondly, the differentially expressed transcripts were identified using LIMMA package in Bioconductor [43]. Thirdly, a heatmap was generated that contains Z-score [expression of X in a sample - (mean expression of X from all samples/standard deviation of X of all samples] which is calculated by the heatmap.2 scale function in R with parameter scale=”row”. Subsequent analysis of gene expression data was done in the freely available statistical computing language R (https://www.r-project.org/) with packages available from the Bioconductor project (https://www.bioconductor.org/). Further pathway analysis was carried out using MetaCore (Clarivate Analytics) and Enrichr [44]. Sequencing data will be made available on a public data repository upon acceptance of the manuscript.

### Cell lines

Human myeloma cell lines H929, MM.1r, and RPMI-8226 were purchased from ATCC and cultured at 37°C, 5% CO_2_ in RPMI-1640 (Invitrogen, A1049101) supplemented with 10% heat inactivated fetal bovine serum (Sigma-Aldrich, F0926). PCNY1 is a clonal cell line derived from a primary patient sample previously described, which is p53 null (17p-; p53 p. Arg248Gly) [9]. PCNY1 cells were cultured at 37°C, 5% CO_2_ in X-VIVO 15 (Lonza, 04-744Q) supplemented with 10% heat inactivated fetal bovine serum (Sigma-Aldrich, F0926) and 1ng/mL IL-6 (R&D, 206-IL). HEK293T cells were purchased from ATCC and cultured at 37°C, 5% CO_2_ in DMEM (Corning, 15-017-CV) supplemented with 10% heat inactivated fetal bovine serum (Sigma-Aldrich, F0926), 1mM sodium pyruvate (Corning, 25-000-CI) and 6 mM L-glutamine (Gibco, 25030-081).

### shRNA lentiviral particles

Lentiviral shRNA particles targeting MAGE-A3 (shMA3) and a scrambled non-target sequence (shNT) were produced following the lentiviral packaging protocol from Sigma-Aldrich (SHP001). Low passage HEK293T cells were transfected with plasmid constructs containing the shRNA sequences targeting MAGE-A3 (TRCN0000128375 and TRCN0000129750, Sigma-Aldrich) or a non-target sequence (SHC002, Sigma-Aldrich). Viral supernatants were harvested, concentrated using Lenti-X concentrator (Clontech, 631231) and titer was obtained by HIV-1 p24 ELISA (Zeptometrix, 0801111). Optimal multiplicity-of-infection (MOI) was determined for each cell line on a lot-to-lot basis.

### Lentiviral transduction of HCMLs

Overnight cultures were harvested and resuspended in growth media supplemented with 8 μg/ml polybrene (Sigma-Aldrich, H9268). Cells were then plated at 10,000 to 40,000 cells per well into a 96-well round bottom plate. Lentiviral particles were added at optimal MOI for each cell line. Cells were incubated for 18 hours at 37°C, then washed twice with growth media to remove excess virus and polybrene. At various timepoints, cells were harvested for immunoblotting, gene expression profiling or treated with chemotherapy drugs.

### Chemotherapy kill curves

Washed cells were resuspended in growth media at 60,000 cells/ml or 150,000 cells/ml for MM.1r and H929, respectively. Resuspended cells were then aliquoted at 100 μl per well into a 96-well flat bottom white plate and incubated at 37°C, 5% CO_2,_ until Bortezomib (SelleckChem, S1013) or Melphalan (MPBio, 155345) treatment time, which was at 24 hrs after transduction for MM.1r and at 48 hrs after transduction for H929. Cells were treated in triplicates at each concentration. Viability was assayed for after 24 hrs of treatment with the CellTiter-Glo Luminescent Cell Viability Assay (Promega, G7572). Viability curves were generated with Prism 6.0 and normalized to vehicle treated controls.

### Immunoblotting

Whole cell lysates were collected by lysing cells in RIPA lysis buffer (50 mM Tris [pH7.4], 150 mM NaCl, 1% NP-40, 0.1% SDS, 1% sodium deoxycholate) containing 1× protease and phosphatase inhibitor (Pierce, 78447). Heavy membrane (HM) fractions were prepared by incubating cells with cold TIB buffer (300 mM treholase, 10 mM HEPES pH 7.4, 1 mM EDTA, 1 mM EGTA, 10 mM KCl, 0.1% BSA) containing 1× protease and phosphatase inhibitor for 20 minutes on ice. Swollen cells were then homogenized by douncing (Wheaton Tissue Grinder, 358003). Unlysed cells were pelleted at 800 xg and discarded. Cytoplasmic supernatant was then collected after centrifugation at 8,000 xg. The remaining HM pellet was lysed with RIPA buffer to obtain the HM fraction. Protein concentration was determined by Bradford assay (Bio-Rad, 500-0205). 10 μg to 15 μg of lysate was run on a SDS-PAGE gel and transferred to a PVDF membrane. Blocking and antibody dilutions were done in 5% non-fat dry milk or 5% bovine serum albumin in TBS-T (20 mM Tris, 140 mM NaCl, 0.1% Tween 20, pH 7.4). The antibodies used were: Bcl-2 (Cell Signaling Technologies, 2872), Bcl-xL (2762), BID (2002), BIM (2819), CoxIV (4850), Mcl-1 (4572), pSer69-BIM (4585), pSer77-BIM (12433), p21 (2947), BMF (Abcam, ab181148), GAPDH (ab8245), Bcl-G (Santa Cruz, sc-393715), Caspase 3 (sc-7272), MAGE6C1 (sc-20034), PUMA (sc-374223), p53 (sc-126), goat anti-mouse IgG HRP conjugate (Thermo Scientific, 32430) and goat anti-rabbit IgG HRP conjugate (32460). Blots were visualized with Supersignal West Pico Chemiluminescent Substrate (34087) or Supersignal West Femto Chemiluminescent Substrate (34096). Relative densities were obtained using ImageJ. Statistical analysis was performed using PRISM 6.0.

## SUPPLEMENTARY MATERIALS



## Abbreviations

ASCT: Autologous stem cell transplantation
CDK: Cyclin-dependent kinase
CTAg: Cancer-testis antigen
DEG: Differentially expressed genes
GEP: Gene expression profiling
HMCL: Human myeloma cell line
MAGE: Melanoma Antigen Gene
MM: Multiple myeloma
RING: Really interesting new gene
RNAi: RNA interference
wt: Wild-type
