# Supplementary material for: MAGE-A inhibit apoptosis and promote proliferation in multiple myeloma through regulation of BIM and p21Cip1
Source: Oncotarget. 2020 Feb 18;11(7):727–39. doi: 10.18632/oncotarget.27488 (PMC7041939; doi:10.18632/oncotarget.27488)
Supplement: Supplementary file 1 [file oncotarget-11-727-s001.pdf]

MAGE-A inhibit apoptosis and promote proliferation in multiple myeloma through regulation of BIM and p21Cip1

SUPPLEMENTARY MATERIALS

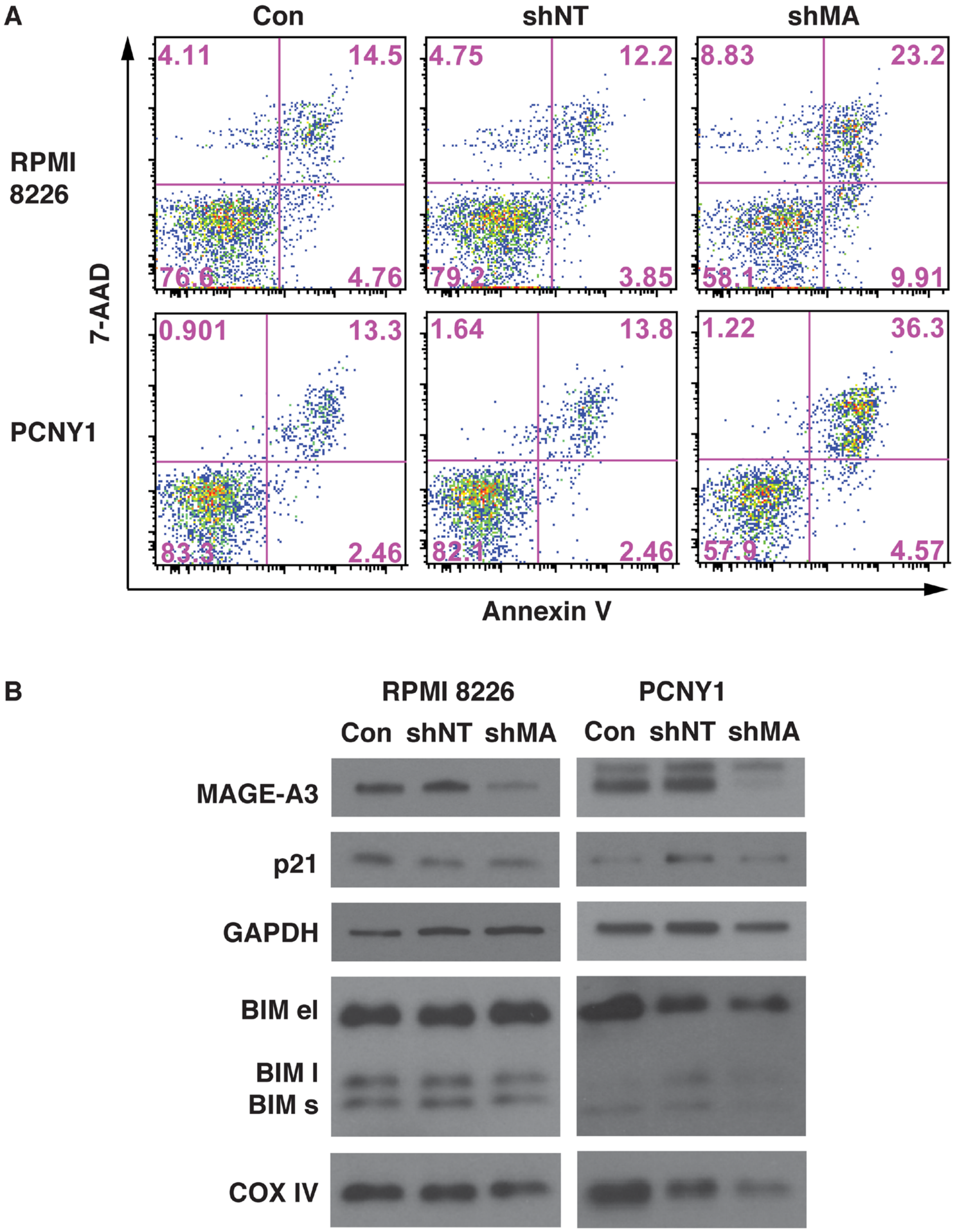

Supplementary Figure 1: Silencing of MAGE-A in p53 null HMCL results in apoptosis but does not affect BIM or p21 protein. (**A**) RPMI8226 and PCNY p53 null MHCL were transduced with MAGE-A or control shRNA lenti as in Figure 1. Apoptosis was assessed at 48 hrs post-transduction by annexin V and 7-AAD staining and flow cytometry. Increased apoptosis, denoted by AnnexinV positive quadrants, was observed in shMA-transduced cells relative to controls. Con, Untreated control cells. shNT, non-target shRNA lentiviral construct. shMA, MAGE-A3 targeted lentiviral shRNA construct TRCN0000128375. (**B**) Western blotting of whole cell lysates for MAGE-A3, p21, and of heavy membrane preparations for BIM demonstrated no significant change in protein levels relative to controls.

Supplementary Table 1: Multiple myeloma cell lines exhibit dependency on MAGE-A3 in functional RNAi screening

Type	Gene/Compound	Dateset	T-Statistic	*P*-Value
gene	**MAGEA3**	Combined RNAi (Broad, Novartis, Marcotte)	-3.99	7.28E-05
gene	**MAGEA9**	Combined RNAi (Broad, Novartis, Marcotte)	3.59	0.000354
gene	**MAGEB10**	RNAi (Novartis)	-4.6	5.68E-06
gene	**MAGEC3**	RNAi (Broad)	3.63	0.000312

Dependencies enriched in multiple myeloma. Analysis of cumulative data from functional RNAi screening of 34 p53 wt and null HMCL (www.depmap.org; Novartis Project DRIVE, Broad, and Marcotte et al. [13–15]) demonstrates highly significant dependency upon MAGE-A3 for survival.

Supplementary Table 2: HMCL included in Novartis, Broad, and Marcotte et al. RNAi screen. Mei, et al.

Depmap Id	Cell Line	Primary Disease	Tumor Type
ACH-000564	KHM1B	Myeloma	Primary
ACH-001094	HTK	Myeloma	
ACH-000057	OPM1	Myeloma	
ACH-000714	KMS11	Myeloma	Primary
ACH-000658	KMS18	Myeloma	Primary
ACH-000763	MM1S	Myeloma	Primary
ACH-000363	SKMM2	Myeloma	Primary
ACH-000889	KMM1	Myeloma	Primary
ACH-000576	KMS27	Myeloma	Primary
ACH-000854	OCIMY5	Myeloma	
ACH-000588	KMS26	Myeloma	Primary
ACH-000626	U266B1	Myeloma	
ACH-000204	LP1	Myeloma	Primary
ACH-000598	KMS21BM	Myeloma	Primary
ACH-000453	MOLP2	Myeloma	Primary
ACH-000419	KMS28BM	Myeloma	Primary
ACH-000193	KARPAS620	Myeloma	Primary
ACH-000426	KMS20	Myeloma	Primary
ACH-000817	RPMI8226	Myeloma	Primary
ACH-000745	MOLP8	Myeloma	Primary
ACH-001043	COLO775	Myeloma	
ACH-001162	PCM6	Myeloma	Primary
ACH-000838	AMO1	Myeloma	Primary
ACH-000050	NCIH929	Myeloma	Metastasis
ACH-000380	KMS12BM	Myeloma	Primary
ACH-000024	OPM2	Myeloma	Primary
ACH-000436	OCIMY7	Myeloma	
ACH-000829	HUNS1	Myeloma	
ACH-000821	EJM	Myeloma	Primary
ACH-000512	INA6	Myeloma	
ACH-000653	JJN3	Myeloma	Primary
ACH-001541	KMS28PE	Myeloma	Primary
ACH-000183	L363	Myeloma	Primary
ACH-000541	KMS34	Myeloma	Primary

Supplementary Table 3: Quantification of Western blot data

	MM.1r			H929		
**Protein**	**shCon**	**shNT**	**shMA**	**shCon**	**shNT**	**shMA**
MAGE-A3	1.08	1.00	0.09	1.30	1.00	0.14
p53	1.20	1.00	3.00	0.70	1.00	4.80
BIM el	1.91	1.00	6.17	0.75	1.00	4.21
BIM l	1.44	1.00	1.87	0.42	1.00	3.40
BIM s	4.40	1.00	10.79	0.48	1.00	1.30
BID	2.09	1.00	1.58	0.34	1.00	0.22
PUMA	1.85	1.00	1.29	1.43	1.00	0.83
Bcl-2	0.91	1.00	0.61	1.11	1.00	0.27
Bcl-xL	1.48	1.00	1.37	0.63	1.00	3.91
Mcl-1 - full length	0.16	1.00	1.03	1.36	1.00	0.86
Mcl-1 - cleaved	0.55	1.00	2.02	0.54	1.00	0.35
p-ser69-BIM	--	--	--	0.85	1.00	3.78
p-ser77-BIM	--	--	--	0.93	1.00	2.57
p21	1.14	1.00	2.68	1.15	1.00	2.02

Densitometry values for Mei, et al, Figure 2.
Western blot data from images depicted in Figure 2 were quantified by optical densitometry using the ImageJ software package.
Results were normalized to the shNT values (1.00).
